# Progression of prostate carcinoma is promoted by adipose stromal cell-secreted CXCL12 signaling in prostate epithelium

**DOI:** 10.1038/s41698-021-00160-9

**Published:** 2021-03-22

**Authors:** Fei Su, Alexes C. Daquinag, Songyeon Ahn, Achinto Saha, Yulin Dai, Zhongming Zhao, John DiGiovanni, Mikhail G. Kolonin

**Affiliations:** 1 The Brown Foundation Institute of Molecular Medicine for the Prevention of Disease, The University of Texas Health Sciences Center at Houston, Houston, TX USA; 2 Division of Pharmacology and Toxicology, College of Pharmacy, The University of Texas at Austin, Austin, TX USA; 3 Livestrong Cancer Institutes, Dell Medical School, The University of Texas at Austin, Austin, TX USA; 4 Center for Precision Health, School of Biomedical Informatics, University of Texas Health Science Center, Houston, TX USA

**Keywords:** Cancer microenvironment, Oncology

## Abstract

Aggressiveness of carcinomas is linked with tumor recruitment of adipose stromal cells (ASC), which is increased in obesity. ASC promote cancer through molecular pathways not fully understood. Here, we demonstrate that epithelial–mesenchymal transition (EMT) in prostate tumors is promoted by obesity and suppressed upon pharmacological ASC depletion in HiMyc mice, a spontaneous genetic model of prostate cancer. CXCL12 expression in tumors was associated with ASC recruitment and localized to stromal cells expressing platelet-derived growth factor receptors *Pdgfra* and *Pdgfrb*. The role of this chemokine secreted by stromal cells in cancer progression was further investigated by using tissue-specific knockout models. ASC deletion of *CXCL12* gene in the *Pdgfr* + lineages suppressed tumor growth and EMT, indicating stroma as the key source of CXCL12. Clinical sample analysis revealed that *CXCL12* expression by peritumoral adipose stroma is increased in obesity, and that the correlating increase in *Pdgfr*/*CXCL12* expression in the tumor is linked with decreased survival of patients with prostate carcinoma. Our study establishes ASC as the source of CXCL12 driving tumor aggressiveness and outlines an approach to treatment of carcinoma progression.

## Introduction

Prostate cancer (PCa) develops resistance to therapy as a result of dynamic interaction with tumor stroma, which is composed of various nonmalignant cell types^1^. Cancer-associated fibroblasts (CAF), the mesenchymal stromal cells (MSC) of tumors, is a population of cells in carcinomas that has stirred controversy^2^. While CAFs have been reported to promote cancer progression^3,4^, the attempts to inactivate them have produced conflicting results^5,6^. The underlying mechanisms of CAF effects are poorly understood^7,8^. CAF play multiple roles, including leukocyte recruitment, extracellular matrix (ECM) remodeling, vascularization, and immunosuppression^9^. A key function of CAF appears to be their ability to induce epithelial–mesenchymal transition (EMT) of carcinoma cells^10^. While the role of EMT in metastatic dissemination is debated^11^, acquisition of the “cancer stem cell” properties and resistance to chemotherapy is undoubtedly a hallmark of EMT^7^. Selective CAF-targeting therapies are lacking, and their development is highly anticipated^12^.

CAF are derived from distinct lineages and are heterogeneous^2,7^. Recent reports provide evidence that CAF are at least in part derived from white adipose tissue (WAT) surrounding the tumor^13–15^. Progression of PCa and other carcinomas is aggravated by obesity^16,17^. Our studies in mouse models have shown that WAT, which becomes inflamed, and fibrotic in obesity, enhances cancer progression irrespective of diet^18^. Peritumoral WAT undergoing remodeling in cancer plays a particularly important role in PCa and several other types of cancer. Our studies indicate that adipose stromal cells (ASC), the MSC of WAT, are expanded in obesity, become mobilized, and migrate to tumors^19^. This process, particularly prominent in obese cancer patients, is linked with poor cancer prognosis^19^. There is accumulating evidence that ASC infiltrating tumors from adjacent WAT depots contribute to the population of CAF^18–20^. The molecular signals through which these adipose-derived CAF promote cancer progression are unclear. ASC are a major source of the ECM that drives tumor desmoplasia^21^ and they also secrete trophic factors that stimulate vascularization^18,20^. Some of the cancer-promoting effects of ASC are contact-dependent^22^. Although roles for ASC in therapy resistance have surfaced^23^, identifying these cells as a prospective drug target^24^, their role in metastatic progression has not been explored. By screening a combinatorial library in vivo, we previously isolated a cyclic peptide WAT7 (sequence CSWKYWFGEC) that homes to ASC in both WAT and tumors^25,26^. We have modified it into a compound targeting ASC, termed D-CAN, which can be used for selective ASC depletion^26,27^. By using this reagent, we recently demonstrated the role of adipose-derived CAF in EMT induction, invasiveness, and chemoresistance of PCa cells^28^, as well as cancer metastasis^29^.

Paracrine angiogenic, immunosuppressive, antiapoptotic, and mitogenic signaling by adipose cells plays an important role in cancer^18–20,30^. However, the exact mechanisms through which ASC and their CAF derivatives promote cancer have remained unknown. We recently reported that a chemokine CXCL12, also known as stromal-cell derived factor 1 (SDF-1), signaling via receptors CXCR4 and CXCR7 and activating tumor cell growth and invasion pathways, is responsible for accelerated prostate tumor growth in obesity^31^. Here, based on clinical and preclinical data showing CXCL12 expression by MSC and ASC, we hypothesized that ASC and ASC-derived CAF are a key source of CXCL12 in tumors. We show that inactivation of CXCL12 in ASC suppresses carcinogenic signaling, tumor growth and EMT. We conclude that ASC/CAF-derived CXCL12 is an important drug target.

## Results

### ASC recruitment in obesity is linked with EMT and CXCL12 expression

**Fig. 1:**
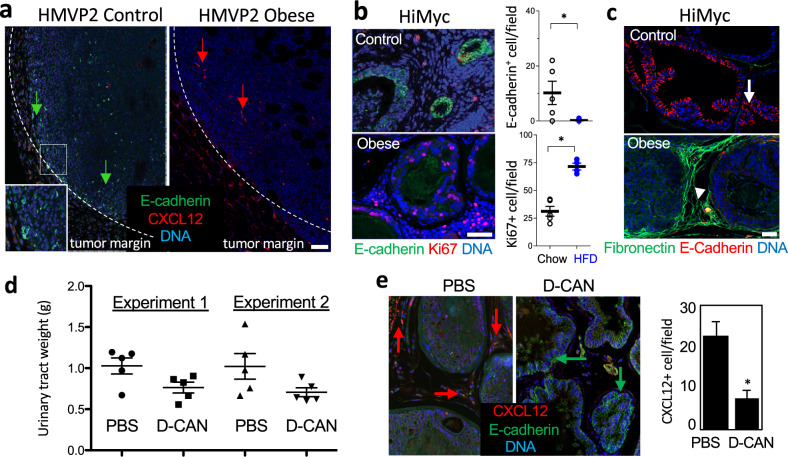
ASC recruitment in obesity is linked with EMT and CXCL12 expression. **a** FVB mice pre-fed low-fat or high-fat diet to induce DIO were grafted with HMVP2 spheroids. Representative resected tumors were analyzed for CXCL12 and E-Cadherin (arrows) in tumor sections. Note membrane E-cadherin (inset: high magnification) and its loss (indicating EMT) concomitant with CXCL12 expression in DIO. **b** IF on sections of ventral prostates of 6-month-old HiMyc mice fed chow (control) or HFD (obese) reveals proliferation (Ki67+) in tumor cells lacking E-cadherin in obesity. Quantification of IF data (based on counts from *N* = 5 view fields) from mice raised on chow and HFD is graphed on the right. **c** IF on sections of ventral prostates of control and obese HiMyc mice (9 months old). E-cadherin in prostate epithelium (arrow) is downregulated in DIO mice concomitantly with fibronectin induction in the stroma (arrowhead). **d** HiMyc mice maintained on HFD treated with D-CAN starting at 12 weeks of age for 4 weeks and terminated at 16 (experiment 1) or 24 (experiment 2) weeks of age have lower urinary tract weight, compared to control mice treated with PBS. *N* = 5. **e** IF analysis of ventral prostate of mice from **d**. Note induction of E-cadherin in epithelium concomitant with reduction of CXCL12 in the stroma, indicating EMT reversal upon D-CAN treatment. Graph: quantification of CXCL12+ cells in the stroma in (**e**); *N* = 5. For all panels, *P < 0.05 compared to control (Student’s *t* test). Scale bar = 100 µm.

To determine the role of ASC in cancer progression, we have used mouse models of PCa, for which the obesity-cancer relationship has been established^31^. We fed mice high-fat diet (HFD; 60 kcal%) and then compared cancer progression in mice with diet-induced obesity (DIO) and control mice raised on 10 kcal% low-fat diet. In FVB mice subcutaneously grafted with HMVP2, a PCa cell line derived from HiMyc mice, obesity was associated with accelerated tumor growth, as reported previously^28^. Importantly, we observed a greater loss of E-cadherin expression, indicating EMT, in tumors of obese mice (Fig. 1a). The EMT was linked with increased immunostaining for CXCL12, a chemokine previously found to promote PCa in obesity and detectable in peritumoral stroma/vasculature but not in adipocytes^31^. To investigate the function of adipose stroma in a genetic model of PCa, we used HiMyc mice, in which over-expression of c-Myc in the prostate is driven by the ARR_2_Pb probasin promoter and results in tumors sharing molecular and histopathological features with human prostate adenocarcinomas^31,32^. At 6 months of age, HiMyc mice raised on HFD displayed accelerated cancer progression, evidenced by significantly higher cell proliferation (Ki67 expression) and earlier loss of E-cadherin expression in prostate glands (Fig. 1b). EMT induction acceleration by obesity was linked with increased fibronectin deposition in the stroma adjacent to prostate cells losing E-cadherin expression, indicating EMT also occurring in this spontaneous model (Fig. 1c).

### Depletion of CXCL12-expressing stroma suppresses EMT

To assess ASC as a source of CXCL12, we analyzed HiMyc mice treated with D-CAN, an ASC-depleting compound, starting at 12 weeks of age. In two independent experiments, we observed an effect of D-CAN on PCa progression, revealed by a trend for lower weight of genitourinary tracts in treated mice (Fig. 1d). Importantly, immunofluorescence (IF) analysis of prostate glands from D-CAN-treated animals demonstrated a significantly lower frequency of CXCL12+ cells in the stroma and a markedly increased E-cadherin expression in the prostate epithelium (Fig. 1e).

**Fig. 2:**
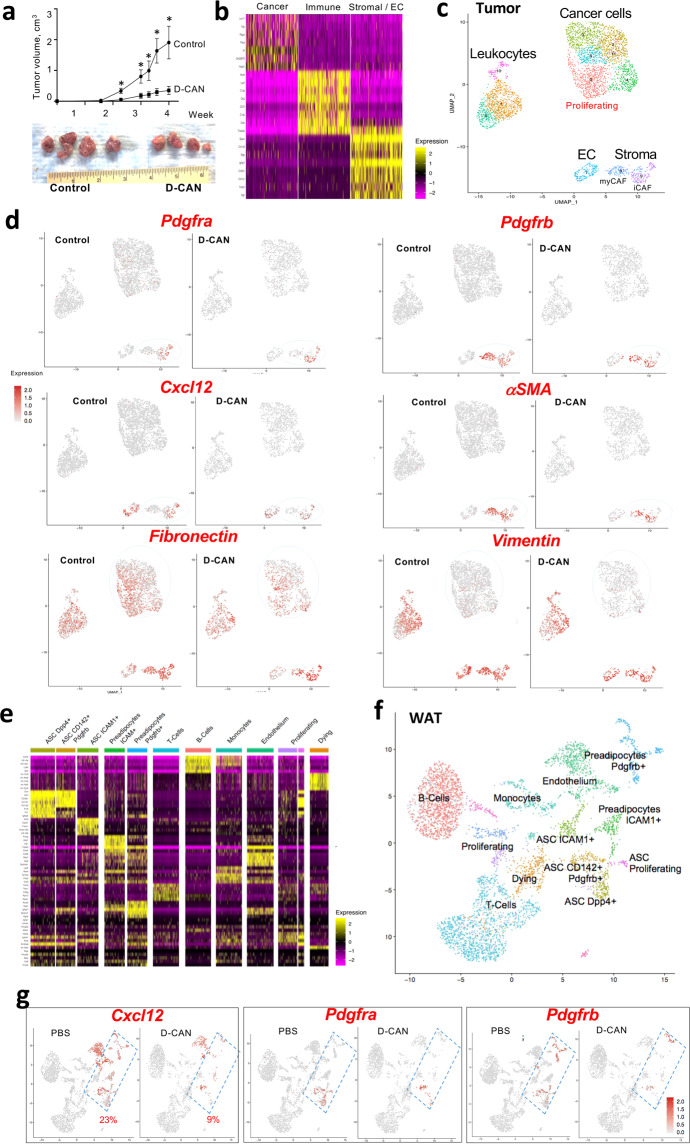
Changes in tumors and microenvironment resulting from D-CAN treatment. **a** Growth of Myc-Cap tumor grafts suppressed by D-CAN treatment in FVB mice fed chow. Graphed is mean tumor volume ± SEM; *N* = 4; *P < 0.05 compared to control (Student’s *t* test). **b** Integrated heatmap of combined tumor scRNAseq data from control and D-CAN-treated mice identifying cell populations expressing genes listed vertically. **c** UMAP clusters of cells identified by scRNAseq in tumors. **d** Co-expression (red) of *Cxcl12, Pdgfra, Pdgfrb*, and *αSMA*, in stromal cells and depletion of the co-expressing sub-population (iCAF) by D-CAN treatment. Note the reduction of *Fibronectin* and *Vimentin* expression in cancer cells. **e** Integrated heatmap of combined SAT scRNAseq data from PBS-injected (control) and D-CAN-treated mice. Selectively expressed genes (left) identify cell clusters designated on top. **f** UMAP clusters of cells identified in SAT cells by combining SAT scRNAseq data from control and D-CAN-treated mice. Proliferating cells are identified based on Ki67 expression, dying cells are identified based on mitochondrial gene expression. Subpopulations of ASC expressing *Dpp4, CD142*, and *Icam1* are indicated. **g** Gene expression in UMAP clusters. Note that D-CAN depletes ASC and preadipocytes (combined with the defined area) co-expressing *Pdgfra, Pdgfrb*, and *Cxcl12*. Frequencies among total SVF are indicated.

To characterize the effect of D-CAN, we performed scRNA-seq analysis. As a model, we used FVB mice fed chow and subcutaneously grafted with MycCaP cells, a cell line derived from HiMyc mice^33^. As in other models, D-CAN significantly suppressed tumor growth (Fig. 2a). Unsupervised hierarchical analysis of cells from tumors identified several subpopulations of cancer cells CD31+ endothelial cells, various CD45+ leukocytes populations, as well as stromal/vascular cells (Fig. 2b). Uniform manifold approximation and projection (UMAP) analysis was then performed. This identified two subpopulations of stromal cells (Fig. 2b), corresponding to myofibroblastic (myCAF) and inflammatory (iCAF) populations of CAFs previously designated in pancreatic cancer models^34^. *Cxcl12, Pdgfra and Pdgfrb* were co-expressed in iCAFs, while only *Cxcl12 and Pdgfrb* were co-expressed in myCAFs (Fig. 2c). Stromal cells were notably depleted by D-CAN treatment (Fig. 2c). D-CAN treatment also reduced the abundance of cancer cells expressing fibronectin and vimentin, reinforcing the notion that stromal ablation results in EMT suppression. Proliferating cells, identified based on Ki67 expression, were also reduced. To compare D-CAN effects on tumor and microenvironment, we also performed scRNA-seq on stromal/vascular factor (SVF) cells of subcutaneous AT (SAT). Unsupervised hierarchical analysis identified endothelial cells and leukocytes populations as well as previously reported^35–37^ CD45−CD31− preadipocytes and several populations of ASC (Fig. 2e). Specifically, we identified the primitive *DPP4*+ ASC, as well as its reported^35^ derivative *CD142*+ ASC and *ICAM1*+ ASC populations by UMAP analysis (Fig. 2f). As shown in Fig. 2g, *Cxcl12, Pdgfra*, and *Pdgfrb* were co-expressed by stromal cells, preadipocytes and the endothelium. As in tumors, comparison of cell populations in control and D-CAN-treated mice demonstrated a marked depletion of all ASC and preadipocyte populations expressing *Pdgfrs* and *Cxcl12* (Fig. 2g).

### *Pdgfra+ and Pdgfrb+* lineages are the source of tumor CXCL12

**Fig. 3:**
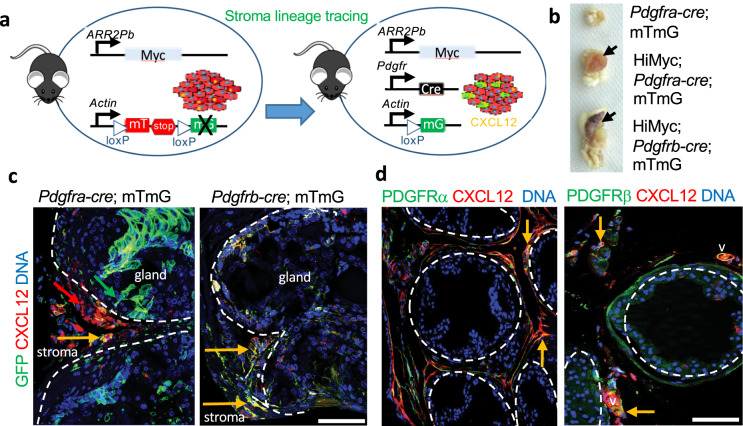
CXCL12 in tumors are expressed by Pdgfr+ lineage stromal cells. **a** scheme for mT (RFP)/mG (GFP) lineage tracing of CXCL12 expression in the stroma with *PDGFRa-Cre and PDGFRb-Cre* drivers in mice. **b** Representative genitourinary tracts from *Pdgfra-cre;mTmG, HiMyc;Pdgfra-cre;mTmG, and HiMyc;Pdgfra-cre;mTmG* mice (C57BL/6 background) fed chow at 12 months of age showing tumors (arrows) in HiMyc+ mice. **c** *PDGFRa-Cre;mTmG* and *PDGFRb-Cre;mTmG* lineage tracing in tumors from HiMyc+ mice fed chow. Cells that have expressed Cre driven by a *PDGFR* promoter, are mG+ (as detected by anti-GFP IF) due to loxP-flanked mT excision (mT fluorescence in other cells is lost in paraffin sections). Yellow arrows indicate that mG+ stroma expresses CXCL12. Tumor epithelium is defined by dashed line. **d** Ventral prostate from HiMyc mice fed chow subjected to IF with indicated antibodies. Note CXCL12 deposits adjacent to and co-localizing with (yellow) stromal cells expressing PDGFRα and PDGFRβ. DNA is blue. Scale bar = 100 µm.

While we previously demonstrated CXCL12 expression in periprostatic WAT^31^, the identity of cells serving as its main source has remained unclear. We and others have reported that MSCs, which can be collectively identified based on the expression of platelet-derived growth factor receptors, PDGFRα (CD140a) and PDGFRβ (CD140b), are heterogeneous. We have reported that PDGFRα+ and PDGFRβ+ can help distinguish subpopulations of stromal cells in WAT and in tumors^26,27^. Recently, we used the *Cre/LoxP* system for ASC lineage tracing in mice and reported that *Pdgfra*+ and *Pdgfrb*+ lineages represent distinct adipocyte progenitor populations^38^. We opted to use this *Pdgfr* tracing approach based on double-reporter *mTmG* system^38^ to identify the lineages of cells expressing CXCL12 in the tumor microenvironment. We generated and analyzed triple-transgenic HiMyc;Pdgfra-Cre;mTmG and HiMyc;Pdgfrb-Cre;mTmG mice (Fig. 3a). Because Pdgfra-cre, and Pdgfrb-cre strains are in the C57BL/6 background, we backcrossed HiMyc mice into the C57BL/6 background for nine generations to prevent genetic heterogeneity in the progeny of crosses between these strains. As previously reported for HiMyc in C57BL/6 background^39^, prostate lesions were histologically evident in over 80% of mice fed chow after 6 months of age and resulted in apparent tumors by 12 months (Fig. 3b). IF with anti-GFP antibodies demonstrated that the majority of stromal cells surrounding PCa cells are traced by *Pdgfra* and *Pdgfrb* promoters (Fig. 3c). Of note, the *Pdgfra* promoter traced some epithelial cells (Fig. 3c). In contrast, *Pdgfrb-cre* selectively traced CXCL12-expressing stroma (Fig. 3C). Our data indicate that *CXCL12* is expressed by both *Pdgfra*+ and *Pdgfrb*+ stromal-cell populations. To confirm which stromal cells express CXCL12, we co-stained prostate tissue of HiMyc+ mice fed chow with CXCL12 and PDGFRα or PDGFRβ antibodies. IF revealed CXCL12 in both PDGFRα+ and PDGFRβ+ cells in tumor stroma, as well is in the adjacent matrix and blood vessels (Fig. 3d).

### CXCL12 knockout in mesenchymal cells suppresses EMT

**Fig. 4:**
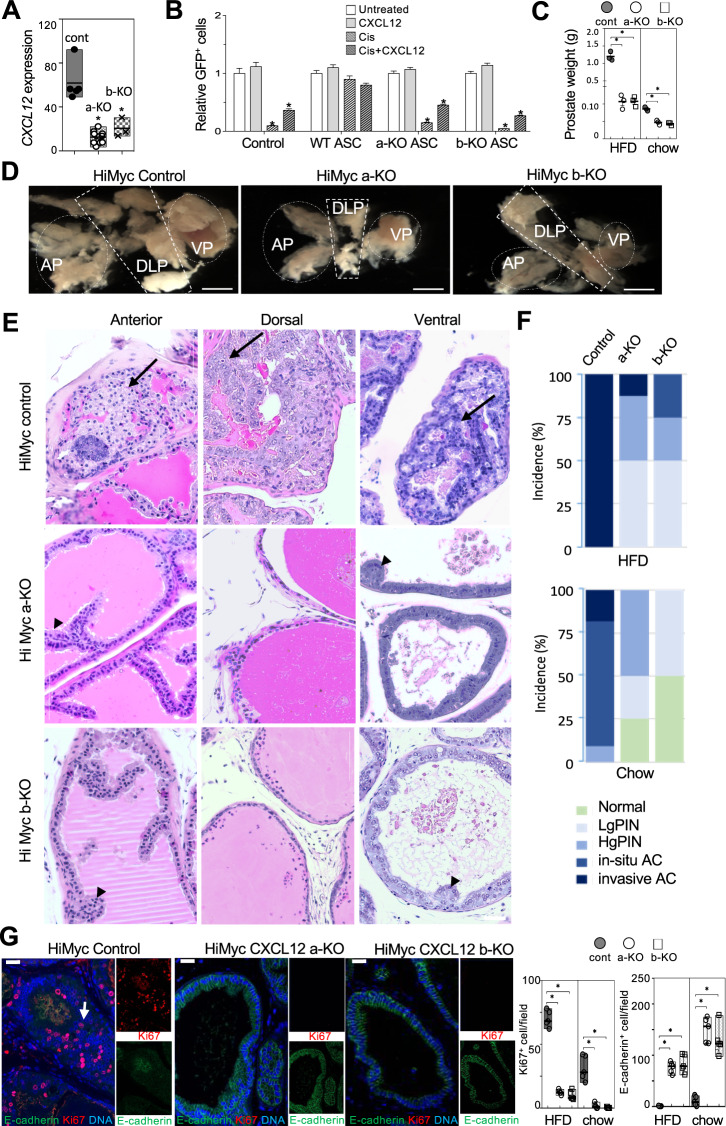
CXCL12 knockout in stroma suppresses tumor growth and EMT. **A** RT-PCR analysis of CXCL12 expression, normalized to *18S RNA*, in periprostatic WAT of 6-month-old control *HiMyc* (cont, *N* = 5), *HiMyc;PDGFRa-Cre;CXCL12*^*flox/flox*^ (a-KO, *N* = 10) and *HiMyc;PDGFRb-Cre;CXCL12*^*flox/flox*^ (b-KO, *N* = 3) mice raised on HFD. *P < 0.01 (Student’s *t* test). **B** Quantification of GFP + LNCaP cells, cultured with ASC from chow-fed *HiMyc*-negative WT, a-KO, and b-KO mice or without ASC (Control) in medium containing 0.2 mg/ml CXCL12 and/or cisplatin (Cis) where indicated. Cell numbers are normalized to “Untreated” for Control and each ASC type. *N* = 5 (independent view field counts); *P < 0.01 (Student’s *t* test) compared to no treatment control. **C** Prostate weights (all lobes combined) in 6-month-old control HiMyc (cont), a-KO HiMyc and b-KO HiMyc mice raised on HFD or chow. *N* = 3 mice; *P < 0.05 (Student’s *t* test). **D** Representative urinary tracts from 6-month-old mice raised on HFD showing lower tumor burden (prostate size) in a-KO HiMyc and b-KO HiMyc mice, compared to control HiMyc mice. Anterior (AP), dorsolateral (DLP), and ventral (VP) prostate lobes are indicated. **E** H&E stainings of indicated prostate lobes from 6-month-old mice raised on HFD. Arrows: adenocarcinoma observed in all lobes of control HiMyc mice. Arrowheads: PIN. **F** Quantification of pathology in prostates of control HiMyc (*N* = 10) and CXCL12 a/b-KO HiMyc (*N* = 4–8) mice raised on HFD versus control HiMyc (*N* = 11) and CXCL12 a/b-KO HiMyc (*N* = 12) mice raised on chow. Graphs show % of prostates with only normal glands and those in which low-grade PIN (LgPIN), high-grade PIN (HgPIN), in situ adenocarcinoma (AC) or invasive AC was detected. **G** IF on sections of ventral prostate of 6-month-old HiMyc mice raised on HFD reveals proliferation (Ki67+) in tumor cells (arrow) lacking E-cadherin. In contrast, a-KO HiMyc and b-KO HiMyc mice have E-cadherin+ epithelium. Quantification of IF data (based on counts from *N* = 5 view fields) from mice raised on chow and HFD is graphed on the right. *P < 0.05 compared to control (Student’s *t* test). Scale bar = 100 µm.

While our results indicate that ASCs secrete CXCL12 in the PCa microenvironment, its expression by other cell types has also been reported^40^. To definitively confirm the key source of CXCL12 in vivo, we proceeded to knockout *CXCL12* in stromal cells of mice using the *Pdgfrb* or *Pdgfra* cre driver lines validated above. For this, we performed crosses to generate cohorts of triple-transgenic HiMyc; Pdgfra-Cre; CXCL12^flox/flox^ (a-KO HiMyc) and HiMyc; Pdgfrb-Cre; CXCL12^flox/flox^ (b-KO HiMyc) male mice. In parallel, we generated Cre-negative CXCL12^flox/flox^; HiMyc (here termed WT HiMyc) and HiMyc-negative CXCL12^flox/flox^ (here termed WT) mice. Finally, these crosses produced HiMyc-negative Pdgfra-Cre; CXCL12^flox/flox^ (a-KO) and HiMyc-negative; Pdgfrb-Cre; CXCL12^flox/flox^ cancer-free (b-KO) littermates. The genotypes of mice were identified by PCR as shown in Supplementary Fig. 1a. RT-PCR on mouse periprostatic WAT demonstrated an expected decrease in CXCL12 mRNA in the a-KO and b-KO mice raised on either chow (Supplementary Fig. 1b) or HFD (Fig. 4A), compared to control HiMyc mice. Both a-KO and b-KO mice developed normally and appeared healthy, consistent with a lack of gross phenotype in mice with CXCL12 knocked-out in mesenchymal progenitors^41^. Histological prostate analysis did not reveal abnormalities in the glands (Supplementary Fig. 1c).

To confirm *CXCL12* deleted from stromal cells as the EMT-enabling factor, we isolated visceral ASC from a-KO and b-KO mice fed chow and tested their ability to promote chemotherapy resistance. While ASC from WT mice blocked LNCaP cell death induced by cisplatin, a-KO and bKO ASC failed to rescue cytotoxicity (Fig. 4B), which was quantified by PI incorporation into the nuclei (Supplementary Fig. 2a). The defect in chemoprotection by a-KO and b-KO ASC was partly rescued by addition of CXCL12 to the medium (Fig. 4B). In the scratch wound healing assay, a-KO and b-KO cells did not notably promote migration of LNCaP cells treated with cisplatin, while CXCL12 promoted migration (Supplementary Fig. 2b). Moreover, while RM1 prostate carcinoma cell grafts were resistant to cisplatin in WT mice, their growth was decreased upon cisplatin treatment in both a-KO and b-KO mice (Supplementary Fig. 2c). Importantly, D-CAN treatment did not further suppress tumor growth in a-KO and b-KO mice (Supplementary Fig. 2c), suggesting that D-CAN works at least in part through abrogating CXCL12 signaling *via* ASC ablation. These data demonstrate the efficacy of CXCL12 deletion in ASC and identify CXCL12 as an ASC-derived chemoresistance-enabling factor.

We then analyzed the effect of CXCL12 deletion on cancer progression. Control HiMyc, a-KO HiMyc and b-KO HiMyc males were placed on chow or HFD to expedite cancer progression and analyzed at 6 months of age, at which HiMyc mice in C57BL/6 background start displaying prostate lesions^39^. Analysis of urinary tracts in 6-month-old mice revealed reduced carcinogenesis in a-KO and b-KO HiMyc mice, on both chow and HFD, as evident from prostate weights (Fig. 4C). All three prostate lobes (anterior, dorsolateral and ventral) were smaller in a-KO and b-KO HiMyc mice, compared to HiMyc controls (Fig. 4D). Consistent with a previous report^39^, histological analysis of prostates demonstrated prostatic intraepithelial neoplasia (PIN) and adenocarcinoma development in in anterior, dorsolateral and ventral prostate lobes of HiMyc controls (Fig. 4E). In situ or invasive adenocarcinoma was observed for 90% of control HiMyc mice on either diet (Fig. 4E and Supplementary Fig. 2d). Consistent with previous reports, cancer progression was promoted by HFD (Fig. 4F). In contrast, while a-KO and b-KO HiMyc mice still developed PIN, adenocarcinoma was not detected at 6 months (Fig. 4E and Supplementary Fig. 2d) or 9 months (data not shown) of age in chow-fed mice. Even on HFD, only 25% of CXCL12 KO mice had adenocarcinomas (Fig. 4F). EMT, prominent in tumors of control HiMyc mice, was also reduced in a-KO or b-KO HiMyc mice fed HFD, as evident from E-Cadherin/Ki67 immunostaining quantification (Fig. 4G).

To exclude the possibility that the decrease in cancer aggressiveness in KO mice was a result of systemic changes that could have resulted from CXCL12 deletion in stromal cells, we performed a systematic analysis of mouse anatomy and physiology. Both a-KO and b-KO mice had normal body size as adults and had visceral and subcutaneous adiposity comparable to that of WT littermates (Supplementary Fig. 3a). To rule out a possibility of the effect of CXCL12 loss on body composition, we also performed EchoMRI analysis. CXCL12 KO mice had normal body weight and their lean and fat body mass was undistinguishable from controls (Supplementary Fig. 3b). Steady state glucose levels were identical in WT and CXCL12 KO HFD-fed groups (Supplementary Fig. 3c) and there was no difference revealed by the glucose tolerance test in fasted mice (Supplementary Fig. 3d). No significant differences were revealed by the cold tolerance test, indicating normal function of brown adipose tissue in CXCL12 KO mice (Supplementary Fig. 3e). Finally, HFD-fed WT and CXCL12 KO mice showed similar energy expenditure, as measured by indirect calorimetry (Supplementary Fig. 3f). Combined, these results argue against a possibility that cancer progression could be affected by indirect effects of stromal CXCL12 loss on mouse energy balance. Our data indicate that ASC-derived CXCL12 promotes cancer progression in vivo.

### Clinical relevance of ASC and CXCL12 in prostate cancer

**Fig. 5:**
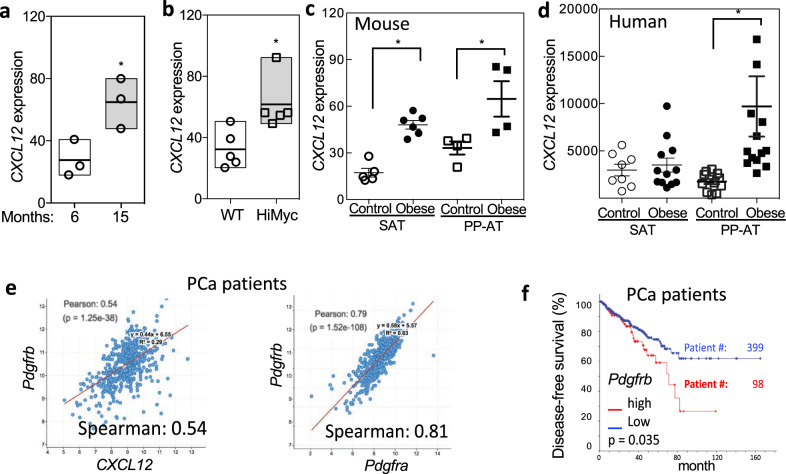
CXCL12 link with age, obesity, and cancer in mice and humans. **a–c** RT-PCR analysis of *CXCL12* mRNA expression normalized to *mGapdh*. **a** Relative *CXCL12* mRNA expression in anterior prostate of 6-month- and 15-month-old HiMyc chow-fed mice; *N* = 3; *P < 0.05 compared to WT (Student’s *t* test). **b** Relative *CXCL12* mRNA expression in anterior prostate of 6-month-old WT and HiMyc chow-fed mice; *N* = 5; *P < 0.05 (Student’s *t* test). **c** Relative *CXCL12* mRNA expression in SAT and periprostatic (PP-AT) adipose tissue of C57BL/6 mice raised for 5 months on chow (control) or HFD (obese); *N* = 4–6; *P < 0.05 (Student’s *t* test). **d** Relative *CXCL12* mRNA expression normalized to *hGapdh* in stromal cells of SAT and periprostatic AT samples from control (BMI < 30; *N* = 8–11) or obese (BMI > 30; *N* = 12–16) PCa patients. *P < 0.05 (Student’s *t* test). **e** Analysis of mRNA expression correlation between *PDGFRb* and *CXCL12*, as well as between *Pdgfrb* and *Pdgfra*, in 498 primary prostate tumors from the TCGA cohort. *P* and *r* values were calculated using Pearson correlation test. **f** Kaplan–Meier curves, based on data from TCGA provisional cohort, showing estimated biochemical recurrence-free probability in patients with high (*z*-score of upper 30%) and low (*z*-score of lower 70%) *PDGFRb* mRNA tumor expression.

Comparison of *CXCL12* mRNA levels in prostate tissue of 6-month and 15-month-old mice revealed an age-dependent increase in expression (Fig. 5a). At 6 months of age, *CXCL12* expression was significantly higher in the prostate tissue of HiMyc mice, compared to WT controls (Fig. 5b). Analysis of WAT stroma demonstrated increased *CXCL12* mRNA levels in obese mice raised on HFD, in particular for periprostatic WAT (Fig. 5c). These results indicate that stromal *CXCL12* expression is induced by age, cancer, and obesity.

**Fig. 6:**
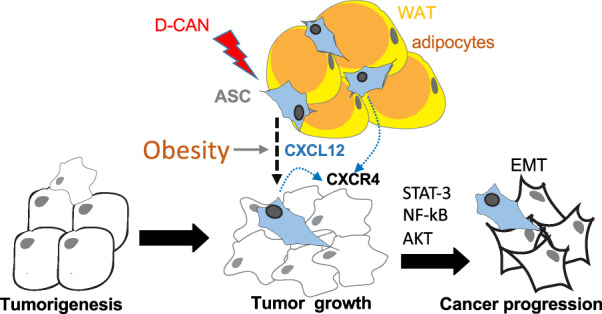
The model of CXCL12 role in cancer progression. Adipose stromal cells (ASC) derived from WAT are the principal source of CXCL12 driving cancer by increasing the survival and proliferation of cancer cells and subsequently their EMT and aggressiveness via CXCR4-mediated signaling. Lightning bolt: D-CAN as a proof-of-principal experimental therapeutic that can be used to deplete ASC and suppress cancer progression.

To address clinical relevance of these observations, we analyzed stroma derived from periprostatic WAT of patients with PCa described previously^19^. We observed that *CXCL12* expression was relatively low in SAT for both lean and obese patients (Fig. 5d). Consistent with observations made in mice, periprostatic WAT *CXCL12* expression in the majority of obese patients was higher than mean concentration observed for lean patients (Fig. 5d). Previous work by us and others has linked obesity with decreased survival of patients with PCa^19^. To assess the potential implication of ASC and CXCL12 in cancer mortality, we analyzed transcriptomic data in 497 PCa patients of the TCGA (provisional) cohort^42^. In the prostate tissue of these patients, there was a correlation between *CXCL12* and *Pdgfrb*, as well as between *Pdgfra* and *Pdgfrb* mRNA levels (Fig. 5e). This suggests that, similar to mice, *CXCL12* is expressed by *Pdgfra*+ and *Pdgfrb*+ stromal cells, abundance of which in the prostate varies among patients. Indeed, analysis of tumors from patients with PCa characterized in our previous study^19^ revealed that prostate tumors contain ASC identified with a peptide probe against the D-CAN receptor (Supplementary Fig. 4). Importantly, progression-free survival is lower in patients with higher *Pdgfrb* (and *CXCL12*) expression (Fig. 5f). Combined, these clinical data suggest that, like the animal models, recruitment of WAT-derived stroma promotes cancer progression, and implicate CXCL12 as a cytokine secreted by ASC that contribute to tumor aggressiveness (Fig. 6).

## Discussion

There is an urgent need for new strategies to effectively suppress PCa resistance to treatment and progression to the advanced stages. Understanding the mechanisms through which stromal cells stimulate carcinoma cells is a prerequisite for development of effective approaches that suppress cancer aggressiveness. It has been reported that CAF promote cancer progression^3,43^. WAT has been identified as a source of CAF^18–20^. In a recent study ASC depletion enhanced chemotherapy efficacy in PCa animal models^28^. However, the role of ASC-derived CAF in PCa virulence has not been explored and the mechanism for the effect of ASC on tumor cells has remained unclear. Here, we investigated the role of ASC in PCa progression. By using genetic PCa mouse models, we show that stromal cells of *Pdgfr* lineages serve as a key source of CXCL12. This study identifies CXCL12 signaling to be rate-limiting for cancer progression in obesity (Fig. 6).

The origins of CAF and their role in cancer progression have remained debated^2^. The heterogeneity of CAF has made it difficult to establish the potential benefits of their inactivation and controversial results have been reported^5,6^. The current challenge is the lack of clarity on the origins and the roles of specific subpopulations of CAF^15^. There are several subpopulations of CAF that have been described: while some CAF have properties of myofibroblasts that produce collagens and drive fibrosis, other CAF specialize in secreting cytokines that shape the tumor immune microenvironment^34^. It has been proposed that CAF can arise from local organ-resident fibroblasts and trans-differentiate from endothelial and epithelial cells^7^. In addition, bone marrow-derived MSC and fibrocytes of myeloid lineage also contribute to CAF^2^. The discovery of WAT as an alternative source of MSC recruited by carcinomas has provided new insights on CAF biology. ASC are composed of subpopulations that can be distinguished by relative *Pdgfra*/*Pdgfrb* expression levels and play different roles in organogenesis^38^. The results presented here show that both *Pdgfra* and *Pdgfrb* lineages contribute to CAF secreting CXCL12. This is consistent with both *Pdgfra*+ and *Pdgfrb*+ cells being derived from *Pdgfra* lineage in development^38^. Our scRNA-seq data further validates this hierarchy and identifies the *Pdgfra*+*Pdgfrb*+ CAF population as the main stromal source of CXCL12. It remains to be reconciled why depletion of *Pdgfrb*+ lineage cells aggravated the progression of pancreatic cancer in mouse models^6^. Because the *Pdgfrb*+ population contains pericytes maintaining vascular integrity, their depletion could be expected to interfere with drug delivery to the tumor. However, in our study ASC depletion potentiated chemotherapy and reduced disease progression, suggesting that the beneficial effect of EMT suppression may outweigh the possible adverse effects of CAF inactivation.

The established role of CXCL12–CXCR4 interaction is to control mobilization of hematopoietic stem cells in the bone marrow^41^. The importance of this chemokine axis in other physiological processes is multifaceted and increasingly appreciated^40^. Accumulating evidence indicates that CXCL12 is pivotal in cancer progression and therapy resistance^31,44,45^. Studies with bone marrow MSC demonstrated their conversion into CAF secreting CXCL12 in response to CXCL16/CXCR6 signaling^43^. It remains to be determined whether this mechanism also underlies the conversion of ASC to CAF. CXCL12 signals through its receptors CXCR4 and CXCR7. Studies in ovarian carcinoma^46^ and our unpublished and published^31^ data indicate that CXCR4 is the main cancer-promoting effector of CXCL12-induced cancer progression. However, we cannot rule out a role for CXCR7 via mechanisms such as heterodimerization with CXCR4^47^. Our previous results based on HMVP2 cell treatment with CXCL12 provide some support for this possibility^31^. In reported meta-analyses, CXCR4 expression has been associated with metastatic disease and poor survival^48,49^. Our results from patient genomic data analysis and animal studies reinforce preceding reports suggesting a link between CXCL12 signaling and EMT in cancer progression to metastases^50^. As we previously reported^31^ in prostate tumors CXCL12–CXCR4 signaling activates STAT3, NFκB, and AKT pathways, which are the likely mechanistic culprits of CXCL12 effects on cancer aggressiveness. Consistent with our results, the contribution of CXCL12 secretion to breast cancer invasiveness has been reported and corroborated by clinical data^51,52^. At odds with these studies, CXCL12 has also been reported to suppress cancer metastasis by regulating CXCR7 receptor expression^53^. Several CXCL12 isoforms have been reported, and their potentially distinct roles in cancer progression remain to be determined. The importance of CXCL12 in PCa progression to metastases has been previously implicated in mouse models. Antimetastatic effects of systemic CXCL12 blockade have been interpreted based on the paradigm that bone marrow MSC attract cancer cells via endocrine CXCL12 signaling^54^. However, systemic CXCL12 circulation in the bloodstream is very low and it has become clear that it mainly acts locally as a paracrine chemokine^40^. Our study, demonstrating that CXCL12 secreted by stromal cells activates invasiveness of adjacent cancer cells, suggests that this chemokine triggers mobilization of cells from the primary tumor and provides an alternative explanation to antimetastatic effects of CXCL12 blockade. Consistent with our results, adipose stroma depletion has been shown to suppress metastasis in breast cancer models^29^. Future studies in metastatic PCa models will help to refine the role of CXCL12 in advanced cancer progression.

Clinical significance of tumor stroma derived from WAT remains to be further established. Obese patients are at a higher risk of disease progression and to chemotherapy/immunotherapy resistance and eventually incurable metastases^16^. The insights into the mechanisms underlying the link between tumor aggressiveness and fat tissue uncovered here may enable new approaches to disease intervention. CXCL12 signaling inhibition synergizes with immune checkpoint blockade by PD-1 and CTLA-4 antibodies in mouse cancer models^44,45^. Further studies will be needed to evaluate WAT-derived CAF as a modulator of immune cell activation in the tumor and a prospective therapy target. Development of compounds targeting CAF or blocking their activity may improve outcomes for patients with disease resistant to chemotherapy and immunotherapy.

## Methods

### Human subjects

The clinical protocol was approved by UT Houston Institutional Review Board. Participants provided written informed consent to take part in the study. Based on the body mass index (BMI; kg/m^2^), subjects were divided into obese (BMI ≥ 30) or lean (BMI < 30). For gene expression analysis, we used mRNA from WAT samples of PCa patients described previously^19^. Freshly isolated (not-plated) SVF from abdominal SAT and periprostatic AT (VAT) were used for mRNA extraction/cDNA isolation and ASC isolation. SAT ASC were from bariatric surgery patients described previously^25^.

### Cell culture and analysis

Cell lines LNCaP (ATCC^®^ CRL-1740)^28^, MycCaP^33^, HMVP2^28,31^, and RM1^19^ have been used as in cited previous studies. Human primary ASC cells from periprostatic WAT^19^ and subcutaneous WAT^38^ were described in the cited previous studies. ASC were grown in EBM2 medium (Lonza).

### Mouse experiments

Studies were approved by and performed according to the guidelines of the Institutional Animal Care and Use Committees of UTHealth and UT Austin. C57BL/6, FVB/N, *mTmG* (Stock 007676), *Pdgfra-Cre* (Stock 013148), *CXCL12*^*flox/flox*^ (Stock 021773) mice were purchased from Jackson Laboratories. *Pdgfrb-Cre* strain was described previously^38,55^. For CXCL12 knockout, HiMyc mice were backcrossed into C57BL/6 background for nine generations prior to crossing with *Pdgfra-Cre, Pdgfrb-Cre, mTmG*, and *CXCL12*^*flox/flox*^ mice. DIO induction and physiological tests were performed as we previously described^27,38^. Subcutaneous grafting of HMVP2 spheroids^28^ and RM1 cells^19^ were as previously described. D-CAN, composed of ASC-homing peptide WAT7 (CSWKYWFGEC) linked *via* aminohexanoic acid with an amphipathic sequence KFAKFAKKFAKFAK^26^ was synthesized from D-amino acids, cysteine-cyclized, and acetate salt chromatographically purified to 99% and quality-controlled (mass spectroscopy) by Ambiopharm. D-CAN was administered subcutaneously as described^26,28^. Tumors were measured with a caliper and volume calculated as length × width^2^ × 0.52.

### Single-cell RNA sequencing^56^^[,57^

Single-cell capture (~3000 cells/sample) and library construction were performed with the Chromium Single Cell 3ʹ Reagent Kit v3.1. Barcoded single-cell gel beads were loaded onto Chromium Next GEM ChipG (PN-1000120). After running on 10× Chromium Single-Cell Controller, gel beads-in-emulsion (GEMs) were generated. The barcoded and full-length cDNAs were produced after incubation of the GEMs and amplified via PCR. Library was qualified by Agilent Bioanalyzer 2100 and quantified by real-time PCR on QuantStudio3. Sequenced was done with Illumina NextSeq 550 System using High Output Kit v2.5 (50,000 reads per cell). The Cell Ranger™ Single-Cell Software Suite v.3.1.0 was used to perform bioinformatic analysis. The reads were aligned to the mouse transcriptome reference (mm10, Ensembl 93) with STAR. Raw read count tables were analyzed using the Seurat (v3.1.1) pipeline on R platform (3.5.2). FindVariableGenes was used to calculate the principal components. Cell clusters were identified using the Shared Nearest Neighbor (SNN) algorithm with a resolution parameter 0.8. UMAP clusters of cells were identified based on the first 10 principal components and feature plots were displayed with the log (raw read count + 1) of gene/cell on UMAP.

### Immunoblotting and immunofluorescence

Tissues were fixed in 10% formalin, paraffin-embedded, and sectioned for IF and H&E staining by histology CORE. Cells from culture were fixed in 4% paraformaldehyde. For IF, performed as described^19,28,31^, primary antibodies were as follows: anti-E-cadherin R&D AF748 (1:100) or Cell Signaling 3195 (1:200); anti-N-Cadherin Abcam ab98952 (1:100) or Cell Signaling 14215 (1:200); anti-CXCL12 sc-28876 (1:200); anti-fibronectin ab23750 (1:200); anti-Ki67 RM-9106-S0 or 14-5698 (1:100) or Cell Signaling 9129 (1:200); anti-PDGFRα ab51875 (1:100); anti-PDGFRβ ab32570 (1:100); anti-GFP GTX26673; 1:250 (1:200). The secondary antibodies were Donkey Alexa 488-conjugated IgG, Cy3-conjugated IgG (Jackson 1:300), Goat Alexa 488-conjugated IgG, Mouse Alexa 594-conjugated IgG (1:500) and Rat Alexa 488 or 594-conjugated IgG (1:500). Nuclei were stained with DAPI. Images were acquired with a confocal Leica TCS SP5 (Leica), Carl Zeiss upright Apotome Axio Imager Z1 or Olympus BX60 fluorescence microscope. Quantification was done with the NIH ImageJ software.

### Reverse transcription PCR

Total RNA was extracted using the Trizol Reagent (Life Technologies, Cat. # 15596018). Complementary DNAs were generated using High Capacity cDNA Reverse Transcription Kit (Applied Biosystems, Cat. # 4368814). PCR reactions were performed on CFX96™ Real-Time System C1000 Touch thermal cycler (Bio-Rad) using Q-PCR Master Mix (Gendepot, Cat. # Q5600-005). The Sybr green primers were as follows: Mouse *Cxcl12*: 5′-TGCATCAGTGACGGTAAACCA-3′, 5′-AGATGCTTGACGTTGGCTCT-3′, human *Cxcl12*: 5′-TTCTTCAGCCGTGCAACAATC-3′, 5′-AGATGCTTGACGTTGGCTCT-3′; *18S RNA*: 5′-AAGTCCCTGCCCTTTGTACACA-3′, 5′-GATCCGAGGGCCTCACTAAAC-3′. Gene expression was normalized to *18S RNA*.

### Statistics

GraphPad Prism or Microsoft Excel were used to graph data as mean ± SEM and to calculate *P* values using homoscedastic Student’s *t* test for most experiments, which were repeated at least twice with similar results. One-way ANOVA was also used to confirm significance for Fig. 4C, G and Supplementary Fig. 2c. To evaluate the relationship between gene expression and disease progression, data from the Cancer Genome Atlas (TCGA) project were downloaded from cBioPortal^42^. Expression data computed as mRNA *z*-scores (log RNA Seq V2 RSEM, Agilent) were compared as we previously described^19^. For Kaplan–Meier survival analysis, the cutoff of 0.7 for stratifying into low and high expression groups was determined using the R2 Genomics Analysis and Visualization Platform method used to reveal significant difference between the cohorts.

### Reporting summary

Further information on research design is available in the Nature Research Reporting Summary linked to this article.

## Supplementary information

Supplementary figures

REPORTING SUMMARY

## Data Availability

The data generated and analysed during this study are described in the following data record: 10.6084/m9.figshare.14039813^56^. The single-cell RNA sequencing data are openly available in the Gene Expression Omnibus repository via the following accession: https://identifiers.org/geo:GSE163701^57^.
