# Supplementary material for: Progression of prostate carcinoma is promoted by adipose stromal cell-secreted CXCL12 signaling in prostate epithelium
Source: NPJ Precis Oncol. 2021 Mar 22;5:26. doi: 10.1038/s41698-021-00160-9 (PMC7985375; doi:10.1038/s41698-021-00160-9)
Supplement: Supplementary file 1 — Supplementary figures [file 41698_2021_160_MOESM1_ESM.pdf]

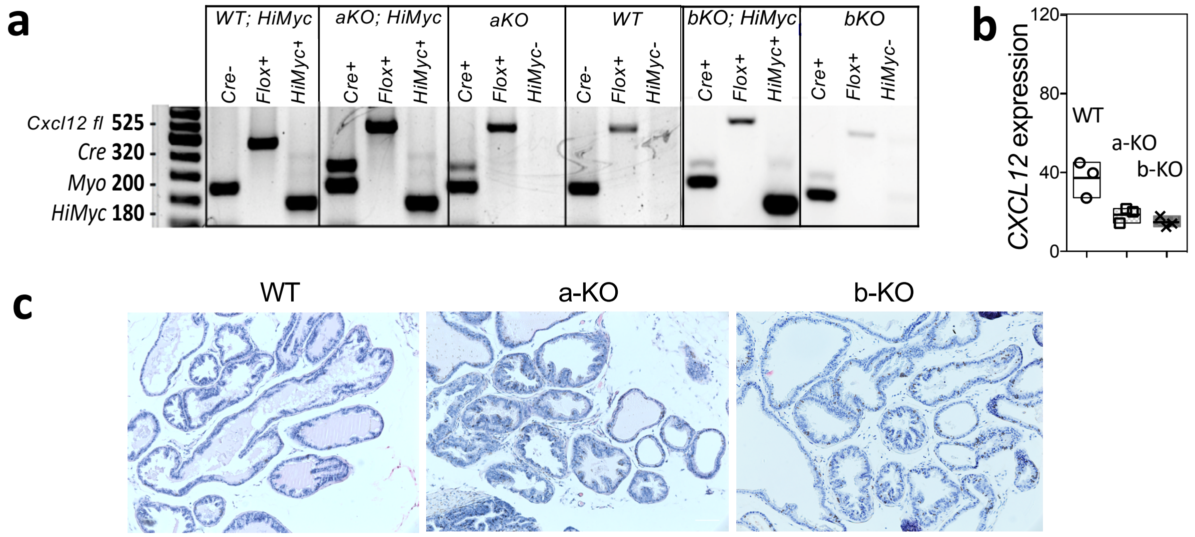

Supplementary Figure 1. Characterization of CXCL12 KO mice. (**a**) PCR genotyping identifying *Pdgfra-Cre;CXCL12*$^{flox/flox}$ (a-KO), *Pdgfrb-Cre;CXCL12*$^{flox/flox}$ (b-KO) and control HiMyc and WT mice. (**b**) RT-PCR analysis of CXCL12 expression, normalized to *18S* mRNA, in periprostatic WAT confirming CXCL12 expression reduction in a-KO and b-KO mice raised on chow. (**c**) H&E stainings of ventral prostates from 6-month-old WT, a-KO and b-KO mice showing normal morphology.

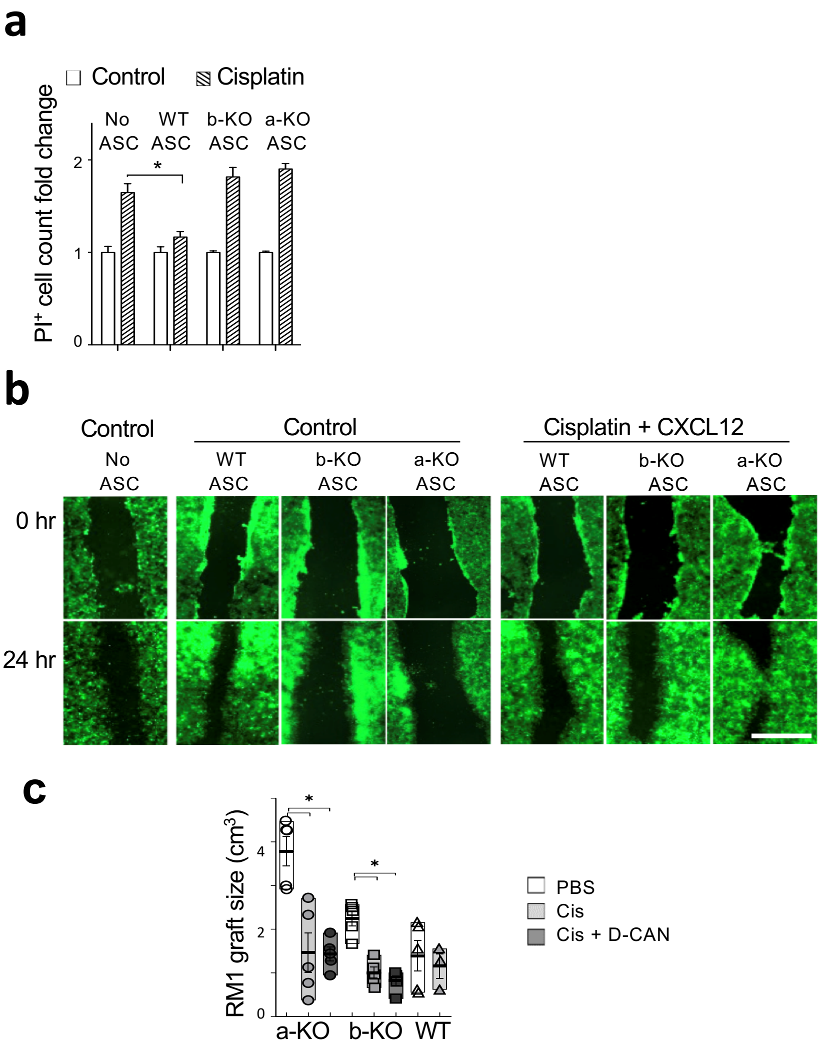

d

Disease Progression in a/b-KO models at 6 months old.					
Diet	Normal	LgPIN	HgPIN	in-situ AC	invasive AC
HFD					
WT	1/10	0/10	0/10	0/10	10/10
a-KO	0/8	4/8	3/8	0/8	1/8
b-KO	0/4	2/4	1/4	1/4	0/4
chow					
WT	0/11	0/11	1/11	8/11	2/11
a-KO	1/4	1/4	2/4	0/4	0/4
b-KO	2/4	0/4	2/4	0/4	0/4

Supplementary Figure 2. ASC secreted CXCL12 confers chemotherapy resistance. (**a**) Killing of GFP+ LNCaP cells, cultured with ASC from WT, a-KO, and b-KO mice or without ASC in medium containing cisplatin. PI incorporation is normalized to control medium for each ASC type. *P<0.01 (Student's t test). ASC from a-KO and b-KO fail to rescue LNCaP cell killing by cisplatin. (**b**) Representative pictures of wound closure in GFP^+ LNCaP monolayer at 0 and 24 hrs after the scratch was made. Wound closure of LNCaP-GFP is promoted by WT ASC but not by a-KO and b-KO ASC co-culture. CXCL12 (200 ng/μl) promotes wound closure of cisplatin-treated LNCaP cells irrespective of ASC co-culture. (**c**) Growth of subcutaneous RM1 grafts in WT, a-KO and b-KO mice (fed chow) treated with 0.6 mg/ml cisplatin +/- D-CAN using the protocol described in Figure 2. Graphed are final graft volumes. N=3-5 / group; *P<0.001. (one-way ANOVA). Note that KO mice are more cisplatin-sensitive with response not further promoted by D-CAN treatment. (**d**) Quantification of pathology in prostates of 6-month-old control and CXCL12 KO HiMyc mice raised on HFD or chow (graphed in Fig. 4f). Shown are numbers of mice with prostates containing only normal glands and those in which low-grade PIN (LgPIN), high-grade PIN (HgPIN), *in situ* adenocarcinoma (AC), or invasive AC was detected.

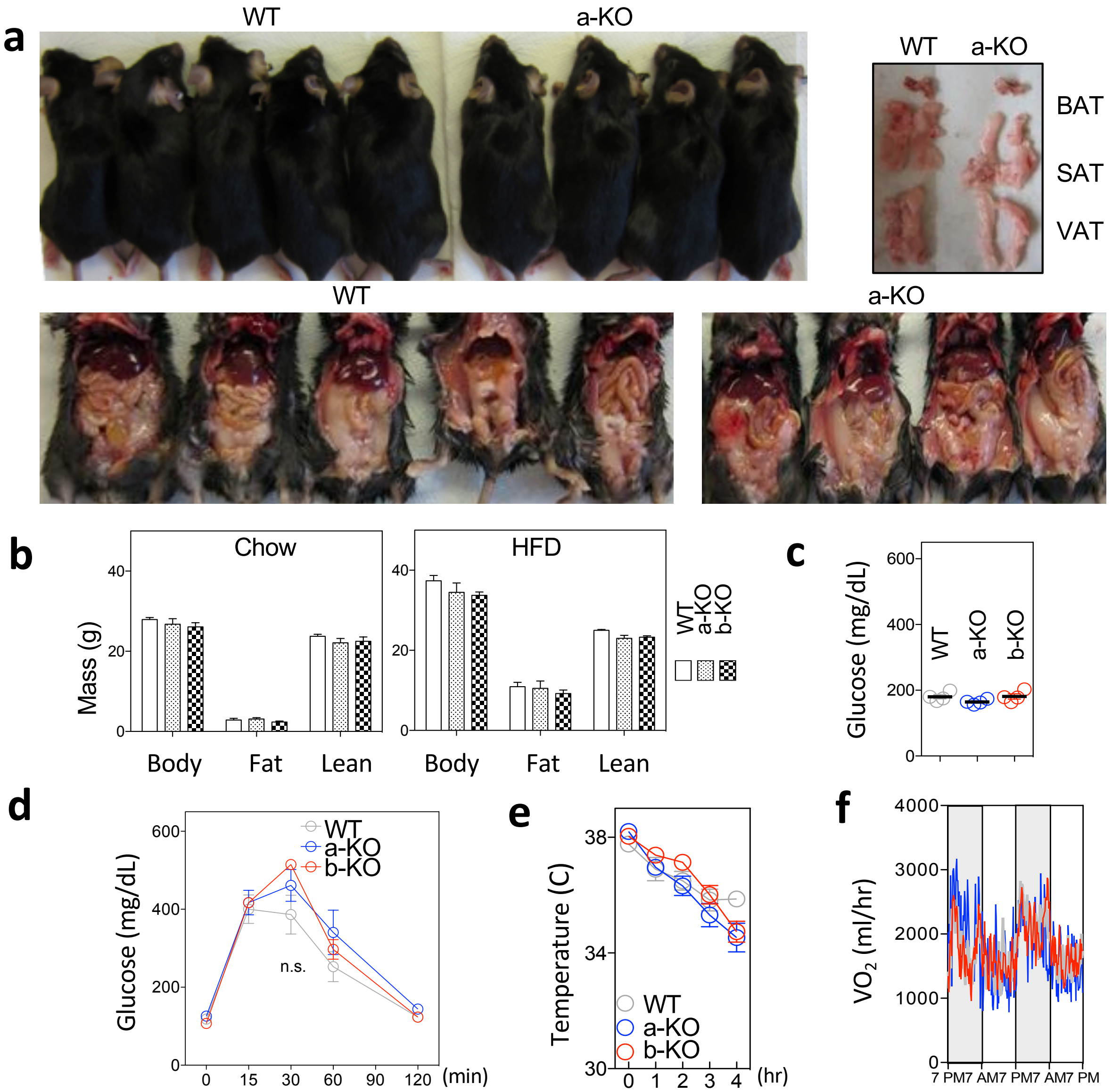

Supplementary Figure 3. Lack of anatomic and physiological abnormalities in CXCL12 KO mice. (**a**) Pictures of WT control and a-KO mice raised on HFD showing comparable adiposity. Top right: resected BAT, SAT and VAT. (**b**) Body composition of WT, a-KO and b-KO mice raised on chow and HFD quantified by Echo MRI. (**c**) Steady state blood glucose levels in WT, a-KO and b-KO mice raised on HFD. (**d**) Glucose tolerance test in fasted WT, a-KO and b-KO mice raised on HFD. (**e**) Cold tolerance in WT, a-KO and b-KO mice raised on HFD. (**f**) Energy expenditure measured by indirect calorimetry over 48 hrs in WT, a-KO and b-KO mice. In all panels, N=4-5 / group.

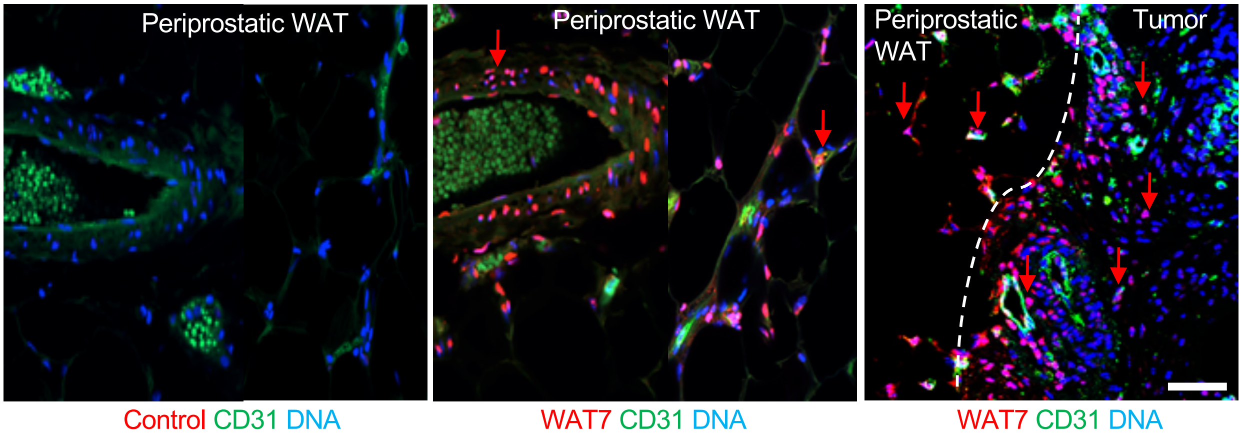

Supplementary Figure 4. D-CAN target expression in human ASC. Peptide WAT7, the ASC-homing domain of D-CAN, was used as a probe to identify ngDCN-expressing cells in sections of human prostate cancer. Binding of biotinylated WAT7 was detected with Cy3-streptavidin (red); CD31 IF (green) marks the endothelium. Note WAT7 (arrows) but not scrambled peptide (control) seen bound to ASC in periprostatic WAT and prostate tumor stroma. Scale bar: 100 µm; Nuclei: blue.
