# Supplementary material for: Progression of prostate carcinoma is promoted by adipose stromal cell-secreted CXCL12 signaling in prostate epithelium
Source: NPJ Precis Oncol. 2021 Mar 22;5:26. doi: 10.1038/s41698-021-00160-9 (PMC7985375; doi:10.1038/s41698-021-00160-9)
Supplement: Supplementary file 2 — REPORTING SUMMARY [file 41698_2021_160_MOESM2_ESM.pdf]

nature research

Corresponding author(s): Mikhail Kolonin

Last updated by author(s): Feb 12, 2021

Reporting Summary

Nature Research wishes to improve the reproducibility of the work that we publish. This form provides structure for consistency and transparency in reporting. For further information on Nature Research policies, see our Editorial Policies and the Editorial Policy Checklist.

Statistics

For all statistical analyses, confirm that the following items are present in the figure legend, table legend, main text, or Methods section.

n/a	Confirmed	
☐	☒	The exact sample size (*n*) for each experimental group/condition, given as a discrete number and unit of measurement
☐	☒	A statement on whether measurements were taken from distinct samples or whether the same sample was measured repeatedly
☐	☒	The statistical test(s) used AND whether they are one- or two-sided<br>*Only common tests should be described solely by name; describe more complex techniques in the Methods section.*
☒	☐	A description of all covariates tested
☒	☐	A description of any assumptions or corrections, such as tests of normality and adjustment for multiple comparisons
☐	☒	A full description of the statistical parameters including central tendency (e.g. means) or other basic estimates (e.g. regression coefficient) AND variation (e.g. standard deviation) or associated estimates of uncertainty (e.g. confidence intervals)
☐	☒	For null hypothesis testing, the test statistic (e.g. *F*, *t*, *r*) with confidence intervals, effect sizes, degrees of freedom and *P* value noted<br>*Give P values as exact values whenever suitable.*
☒	☐	For Bayesian analysis, information on the choice of priors and Markov chain Monte Carlo settings
☒	☐	For hierarchical and complex designs, identification of the appropriate level for tests and full reporting of outcomes
☒	☐	Estimates of effect sizes (e.g. Cohen's *d*, Pearson's *r*), indicating how they were calculated

Our web collection on statistics for biologists contains articles on many of the points above.

Software and code

Policy information about availability of computer code

Data collection	No software was used
Data analysis	Microsoft Excel 16.16.26; Graphpad Prism 7.05/e; ZEN2 Core; ImageJ / Java 1.8.0_172; Amira v2020.2; Quantstudio 3; STAR v2.1.3; Cell Ranger v.3.1.0; Seurat v3.1.1 (R package); R Programming (3.5.2).

For manuscripts utilizing custom algorithms or software that are central to the research but not yet described in published literature, software must be made available to editors and reviewers. We strongly encourage code deposition in a community repository (e.g. GitHub). See the Nature Research guidelines for submitting code & software for further information.

Data

Policy information about availability of data

All manuscripts must include a data availability statement. This statement should provide the following information, where applicable:

- Accession codes, unique identifiers, or web links for publicly available datasets
- A list of figures that have associated raw data
- A description of any restrictions on data availability

Single cell RNA sequencing GEO deposition: GSE163701. Other data that support the findings of this study are available from the corresponding author upon request. There are no restrictions on data availability.

Field-specific reporting

Please select the one below that is the best fit for your research. If you are not sure, read the appropriate sections before making your selection.

☒ Life sciences ☐ Behavioural & social sciences ☐ Ecological, evolutionary & environmental sciences

For a reference copy of the document with all sections, see nature.com/documents/nr-reporting-summary-flat.pdf

Life sciences study design

All studies must disclose on these points even when the disclosure is negative.

Sample size	For animal experiments, N=5 mice (or more) / group with experiments repeated twice with similar result is what we adhered to for experiments done in main display items. These numbers are standard in mouse research in this field / with the methods used.
Data exclusions	There were no data exclusions.
Replication	Animal experiments were repeated at least twice with similar result
Randomization	Allocation was random.
Blinding	Investigators analyzing the data were blinded to data allocation performed by another investigator.

Reporting for specific materials, systems and methods

We require information from authors about some types of materials, experimental systems and methods used in many studies. Here, indicate whether each material, system or method listed is relevant to your study. If you are not sure if a list item applies to your research, read the appropriate section before selecting a response.

Materials & experimental systems

n/a	Involved in the study	
☐	☒	Antibodies
☐	☒	Eukaryotic cell lines
☒	☐	Palaeontology and archaeology
☐	☒	Animals and other organisms
☐	☒	Human research participants
☐	☒	Clinical data
☒	☐	Dual use research of concern

Methods

n/a	Involved in the study	
☒	☐	ChIP-seq
☒	☐	Flow cytometry
☒	☐	MRI-based neuroimaging

Antibodies

Antibodies used	Anti-E-cadherin R&D AF748; anti-N-Cadherin Abcam ab98952 ; anti-CXCL12 sc-28876; anti-fibronectin ab23750; anti-Ki67 RM-9106-S0, 14-5698 and Cell Signaling 9129 ; anti-PDGFRα ab51875; anti-PDGFRβ ab32570 ; anti-GFP GTX26673.
Validation	Each of these antibodies are routinely used in the field. Each has been extensively used and validated by numerous studies by our group and others. This information is available online from the manufacturers for each.

Eukaryotic cell lines

Policy information about cell lines

Cell line source(s)	LNCaP , HMVP2, RM1, MycCap
Authentication	ATCC, cell culture, tumor growth assays
Mycoplasma contamination	All lines are negative for mycoplasma
Commonly misidentified lines (See ICLAC register)	N/A

Animals and other organisms

Policy information about studies involving animals; ARRIVE guidelines recommended for reporting animal research

Laboratory animals	C57BL/6 background mice, both sexes, various ages
Wild animals	No wild animals involved.
Field-collected samples	No field-collected animals involved.
Ethics oversight	UTHealth Animal Care and Use Committee

Note that full information on the approval of the study protocol must also be provided in the manuscript.

Human research participants

Policy information about studies involving human research participants

Population characteristics	Males of various ages were included as published in our previous work Zhang T, et al. and Kolonin CXCL1 mediates obesity-associated adipose stromal cell trafficking and function in the tumor microenvironment. Nature Comm. 2016;7:11674-90.
Recruitment	Described in our publication Zhang T, et al. and Kolonin CXCL1 mediates obesity-associated adipose stromal cell trafficking and function in the tumor microenvironment. Nature Comm. 2016.
Ethics oversight	MD Anderson Cancer Center

Note that full information on the approval of the study protocol must also be provided in the manuscript.

Clinical data

Policy information about clinical studies

All manuscripts should comply with the ICMJE guidelines for publication of clinical research and a completed CONSORT checklist must be included with all submissions.

Clinical trial registration	N/A
Study protocol	Not a clinical trial
Data collection	Adipose tissue samples were collected during prostatectomy.
Outcomes	Outcomes were not a part of the study.
